# Risk factors for venous thromboembolism induced by prolonged bed rest during interstitial brachytherapy for gynecological cancer: a retrospective study

**DOI:** 10.1186/s13014-021-01840-3

**Published:** 2021-06-29

**Authors:** Keiko Nemoto Murofushi, Tetsuya Tomita, Kayoko Ohnishi, Kei Nakai, Azusa Akiyama, Tsukasa Saida, Toshiyuki Okumura, Katsuyuki Karasawa, Toyomi Satoh, Hideyuki Sakurai

**Affiliations:** 1 Department of Radiation Oncology and Proton Medical Research Center, Faculty of Medicine, University of Tsukuba, 1-1-1 Tennodai, Tsukuba, Ibaraki 305-8576 Japan; 2 Division of Radiation Oncology, Department of Radiology, Tokyo Metropolitan Cancer and Infectious Diseases Center Komagome Hospital, 3-18-22 Honkomagome, Bunkyo-ku, Tokyo, 113-8677 Japan; 3 Department of Radiology, School of Medicine, International University of Health and Welfare, 4-3 Kozunomori, Narita, Chiba 286-8686 Japan; 4 Department of Diagnostic and Interventional Radiology, University of Tsukuba Hospital, Amakubo 2-1-1, Tsukuba, Ibaraki 305-8576 Japan; 5 Department of Obstetrics and Gynecology, Faculty of Medicine, University of Tsukuba, 1-1-1 Tennodai, Tsukuba, Ibaraki 305-8575 Japan

**Keywords:** Gynecological cancer, Image-guided adaptive brachytherapy, Interstitial brachytherapy, Venous thromboembolism, Deep vein thrombosis

## Abstract

**Background:**

Screening and management of venous thromboembolism (VTE) after surgery is important in preventing sublethal VTE. However, the risk factors for VTE during interstitial brachytherapy (ISBT) remain unknown, and appropriate screening and management strategies are yet to be established. Therefore, this study aimed to evaluate the risk factors for VTE resulting from requisite bed rest during ISBT for gynecologic cancers.

**Methods:**

We retrospectively analyzed 47 patients. For patients without definitive preceding radiotherapy, whole pelvic irradiation (30–50 Gy) followed by ISBT of 12–30 Gy/2–5 fx/1–3 days was administered to CTV D90. For patients with preceding radiotherapy, 36–42 Gy/6–7 fx/3–4 days was delivered by ISBT alone. The supine position was required during ISBT. D-dimer (DD) was measured at initial presentation, 1 week before ISBT, pre-ISBT, on the day of, and the day following needle removal. Patients were divided into three groups according to the risk of VTE and were managed accordingly; Group 1: DD was not detected (negative) before ISBT, Group 2: VTE was not detected on venous ultrasound imaging, although DD was positive before ISBT, and Group 3: VTE was detected (positive) before ISBT. An intermittent pneumatic compression device was used during ISBT; for the patients without VTE before ISBT. Heparin or oral anticoagulants were administered to patients with VTE before ISBT.

**Results:**

Overall, the median values of DD pre-ISBT, on the day of, and on the day following needle removal were 1.0 (0.4–5.8), 1.1 (0.5–88.9), and 1.5 (0.7–40.6) μg/mL, respectively. After ISBT, no patients had deep vein thrombosis (DVT) in groups 1 and 2. In group 3, 7 of 14 patients experienced worsening of VTE but remained asymptomatic. In univariate analysis, DVT diagnosed before ISBT, Caprini score ≥ 7, and difference in DD values between pre-ISBT and the day of or the day following needle removal ≥ 1 were associated with the incidence or worsening of VTE.

**Conclusion:**

DD should be measured before and after ISBT to detect the incidence or worsening of VTE in patients with DVT. The Caprini score may help in the prediction of VTE during or after ISBT.

## Background

Silent or subclinical venous thromboembolism (VTE) before treatment occurs in 6.7–23.6% of gynecological cancer patients [1–3]. Abdominal and pelvic surgery, or a prior history of VTE, is associated with the incidence of VTE following surgery [4]. D-dimer (DD), which is a degradation product of fibrin and is reflective of fibrin concentration, is useful for the screening of VTE [5, 6]. Geerts et al. reported that pulmonary embolism (PE) was observed in 30% of patients with deep vein thrombosis (DVT), and one-third of the cases of PE were fatal [7]. The screening and management of VTE following surgery plays an important role in the prevention of sublethal VTE. Hence, the American College of Chest Physicians Evidence-based Clinical Practice Guidelines recommends the use of thromboprophylaxis for the occurrence of VTE after surgery, according to the risk classification of the patient [4]. The risk stratification for VTE following surgery is primarily based on the Rogers score, which consists of patient factors, preoperative laboratory values, and operative characteristics [8], and/or the Caprini score, which consists of patient factors and operative characteristics [9].

Interstitial brachytherapy (ISBT) can deliver an adequate dose for the treatment of irregularly-shaped cervical cancer, such as severe vaginal invasion or asymmetrical tumors, compared to intracavitary brachytherapy (ICBT), resulting in good local control (LC) [10]. American brachytherapy society recommended ISBT for these complex situations instead of ICBT [11]. Unlike ICBT, given that 2–3 days of bed rest are necessary to perform ISBT, patients may be more likely to develop VTE during ISBT. However, the risk factors of VTE during ISBT are unknown, and appropriate screening and management has yet to be established. The present study sought to analyze the incidence of VTE during ISBT and to determine the risk factors for VTE in gynecological cancer patients treated with ISBT.

## Methods

### Patient characteristics

A total of 52 patients with gynecological cancer received high dose rate (HDR) ISBT between June 2017 and December 2019 at (blinded for review). We included 47 patients per the following criteria: (1) the screening and management of VTE in this study was adhered; (2) atrial fibrillation was not observed; (3) anticoagulant was not administered for cardiovascular disease, cerebrovascular disease, or other reasons besides VTE. The protocol for this study was approved by the institutional review board at (blinded for review) (IRB No. H30-302), and informed consent was waived due to the retrospective nature of the study.

### Measurement of plasma D-dimer levels

Peripheral blood samples were collected from all patients at initial presentation, 1 week before ISBT, pre-ISBT (0–3 days before needle insertion), on the day of needle removal, and the day following needle removal. Blood samples were collected from an antecubital vein into plastic tubes. 0.11 M sodium citrate was added to whole blood in a 9:1 dilution as an anticoagulant, and citrated plasma was then separated by centrifugation at 3000 rpm for 10 min. Plasma DD levels were measured using nanopia DD (Sekisui Medical, Tokyo, Japan) sensitized with anti-DD mouse monoclonal antibodies to induce a latex coagulation reaction. Turbidity was then quantified by spectrophotometry using the CP3000 automated coagulation system (CP3000; Sekisui Medical, Tokyo, Japan). The cutoff value for the measurement of plasma DD was 1.0 µg/mL.

### Detection of VTE

Venous ultrasound imaging (VUI) was performed to detect deep vein thrombosis (DVT). Ultrasonography was performed using Aplio500 or Aplio a 450 (Canon Medical Systems Corporation, Otawara, Japan) with a 3.5-MHz convex and a 7.5-MHz linear transducer. Power, pulse repetition frequency, and wall thump filter settings were adjusted for venous vascular studies. Femoral, popliteal, peroneal, anterior and posterior tibial, and soleal veins were evaluated bilaterally. Femoral veins were assessed in a supine position, and other veins were assessed in a sitting position. All veins were imaged on transverse and long-axis views. Venous lumina were observed while searching for thrombus by manual compression with transducer and color Doppler imaging (Thrombus was searched by transducer compression and color Doppler imaging). For the patients, who had a DVT on the proximal side beyond the popliteal fossa, intrapelvic DVT and PE were evaluated by contrast-enhanced CT.

### Screening and management of patients with VTE at peri-ISBT period

**Fig. 1:**
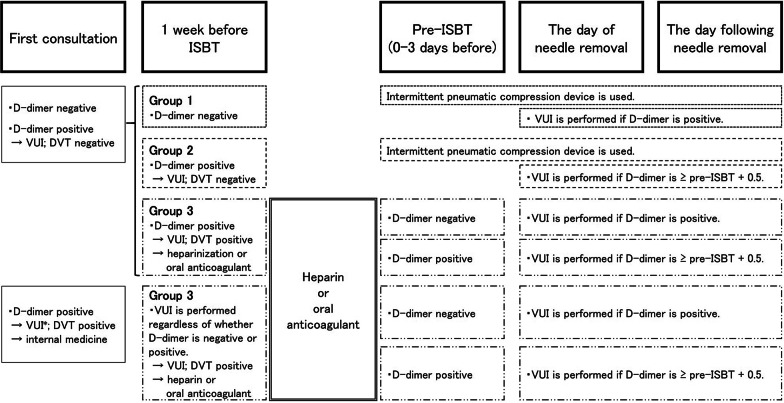
Screening and management of venous thromboembolism. *ISBT* interstitial brachytherapy, *VUI* venous ultrasound imaging, *DVT* deep venous thrombosis

DD was measured in all patients at first presentation, 1 week before ISBT, pre-ISBT, on the day of needle removal, and on the day following needle removal. Patients were divided into three groups based on the risks and management of VTE during the peri-ISBT period (Fig. 1). Patients in Group 1 were negative for DD (< 1.0 µg/mL) 1 week before ISBT. In this group, an intermittent pneumatic compression device (IPCD) was used during the supine position, and VUI (± CT) was performed if DD levels exceeded a standard value of 1.0 µg/mL on the day of or the day following needle removal. In Group 2, VTE was not detected in VUI (± CT), although patients were positive for DD (≥ 1.0 µg/mL) 1 week before ISBT. IPCD was used during the supine position in this group. VUI (± CT) was performed if DD levels on the day of or the day following needle removal exceeded the pre-ISBT value + 0.5 µg/mL. In Group 3, VTE was detected at first presentation and/or 1 week before ISBT. A graduated compression stocking was used during ISBT to maintain the supine position for patients in this group. VUI (± CT) was performed if DD on the day of or the day following needle removal was positive in patients who had negative DD levels pre-ISBT or exceeded the pre-ISBT value + 0.5 µg/mL in patients that had a positive DD level pre-ISBT.

The patients with DVT at first presentation received VUI 1 week before ISBT. Patients with acute or subacute DVT detected by VUI 1 week before ISBT or with PE detected by enhanced CT before ISBT were given unfractionated heparin (UFH), which was subsequently interrupted at midnight on the day of needle insertion. Oral anticoagulant was interrupted 2 days before ISBT for patients with chronic DVT. A bolus injection of 5000 units (80–100 unit/kg) of UFH was initially administered, followed by continuous administration of 750–1000 units/h (15–20 units/kg/h) under monitoring for the activated partial thromboplastin time (aPTT) (target value: 1.5–2.5 × normal value).

### Radiotherapy and brachytherapy protocol

Patients that did not receive preceding radiotherapy underwent external beam radiotherapy (EBRT) and HDR ISBT. The EBRT was delivered using the three-dimensional conformal technique with a linear accelerator (Clinac iX; Varian Medical System, Palo Alto, CA) and a 10-MV photon beam. Whole-pelvic EBRT was initially administered at a dose of 30.0–50.0 Gy in 15–25 fractions using the four-field technique, and an additional dose of 0–20.0 Gy in 0–10 fractions was administered using EBRT with a 3-cm midline block (MB) and the anterior–posterior/posterior-anterior technique [12]. In cases where the shortest diameter of a pelvic lymph node was ≥ 1 cm, a boost EBRT dose of 6–10 Gy was administered in 3–5 fractions.

Brachytherapy was performed using an iridium-192 (^192^Ir) remote afterloading system (MicroSelectron HDRTM; Nucletron, Veenendaal, The Netherlands), and the first HDR ISBT treatment was performed within 7 days after the MB insertion. For patients that did not undergo preceding radiotherapy, ISBT was delivered at a dose of 12–30 Gy in 2–5 fractions on 1–3 consecutive days (prescribed as D90 to high-risk clinical target volume [HRCTV]). For the patients that did receive preceding radiotherapy, ISBT was delivered at a dose of 36–42 Gy in 6–7 fractions on 3–4 consecutive days. ISBT was planned based on CT with a 2.5-mm slice thickness and either the Oncentra system (version 4.0, Nucletron). The HRCTV included the macroscopic residual tumor, which was imaged as the high T2WI area on the pre-brachytherapy magnetic resonance imaging, and the entire cervix [13]. During ISBT, the Martinez Universal Perineal Interstitial Template was used for all patients. Spinal anesthesia and epidural anesthesia were the principal methods of anesthesia for ISBT. In cases where epidural anesthesia was difficult to perform, intravenous patient-controlled anesthesia was used. Bed rest in the supine position was maintained for all patients during ISBT. From the time of needle removal to the next morning, the standing position was prohibited, and bed rest was maintained.

### Chemotherapy

During the radiotherapy period, a weekly regimen of cisplatin (CDDP; 40 mg/m^2^) was generally administered for the patients receiving both EBRT and ISBT. Concurrent chemotherapy with radiotherapy (CCRT) was not performed for patients who met any of the following conditions: (1) ISBT alone, (2) preceding radiotherapy, (3) insufficient renal function, or (4) ≥ 75 years old. A total of 14 patients ultimately received radiotherapy alone, and 33 patients received CCRT.

### Statistical analysis

The incidence of VTE was defined as a newly appearing thrombosis within a specific region where VTE was not present before ISBT. The worsening of VTE was defined as a thrombosis that was larger than the thrombosis observed on VUI or CT before ISBT.

For the patients receiving EBRT, the equivalent dose in 2 Gy fractions (EQD2) was calculated to sum the dose of ISBT plus EBRT before the insertion of the MB, based on the linear-quadratic model [14]. For the patients treated with ISBT alone, the EQD2 was calculated to determine the dose of ISBT. The tumor dose was calculated using an α/β ratio of 10 Gy. To calculate the dose-volume parameters of the organ at risk (OAR) (i.e., D2.0 cc), the α/β ratio was assumed to be 3 Gy.

The paired *t*-test was used to compare DD values between pre-ISBT levels and levels on the day of and the day following needle removal. The difference between pre-ISBT DD values and those on the day of needle removal was determined by subtracting the pre-ISBT DD value from the DD level on the day of needle removal. The difference between pre-ISBT DD values and those on the day following needle removal was determined by subtracting the pre-ISBT DD value from the DD level on the day following needle removal. The Student *t*-test was used to compare these differences. The risk factors for incidence or worsening of VTE and/or PE caused by prolonged bed rest during ISBT were assessed by the Chi-square test. The chi-square test was performed using the SPSS Base System software program version 24.0.0.0 (SPSS, Chicago, IL) and the SAS software version 9.4 (SAS Institute, Cary, NC), and differences were considered statistically significant at *p*-values of <0.05.

## Results

The patients’ characteristics are shown in Table 1. The median age was 61 years. Thirty-three patients received definitive radiotherapy, including ISBT as an initial treatment. Recurrent tumors were present in 14 patients. Of these, 11 patients had a postoperative recurrence, and the remaining three patients experienced recurrence after definitive radiotherapy. Six of the 14 patients received preceding radiotherapy. Thirty-seven of 47 patients (79%) had cervical cancer. The patients had neither a family history of DVT/PE nor a history of DVT/PE before the diagnosis of their gynecological cancer.

**Table 1:** Patient and tumor characteristics

Median age (years)	61 (range 30–86)
Median body mass index (kg/m^2^)	22.0 (range 14.1–37.6)
Primary	
Cervix	37 (79%)
Corpus	7 (15%)
Vagina	2 (4%)
Vulva	1 (2%)
Histology	
Squamous cell carcinoma	36 (77%)
Adenocarcinoma	11 (23%)
Median tumor size, mm	62 (range 9–140)
Initial onset/recurrence	
Initial onset	33 (70%)
Recurrence	14 (30%)
FIGO for 33 patients with initial onset	
III	18 (55%)
IVA	15 (45%)
PLN metastasis at the time of pre-radiotherapy	
Negative	17 (36%)
Positive	30 (64%)
PALN metastasis at the time of pre-radiotherapy	
Negative	35 (74%)
Positive	12 (26%)
Median Caprini score at the time of pre-ISBT	5 (range 3–10)
Median Rogers score at the time of pre-ISBT	4 (range 1–5)

*FIGO* International Federation of Gynecology and Obstetrics, *PLN* pelvic lymph node, *PALN* para-aortic lymph node, *ISBT* interstitial brachytherapy

Treatment characteristics are shown in Table 2. A total of 41 patients received EBRT plus ISBT, and ISBT alone was performed on six patients with preceding radiotherapy. The median duration of bed rest was 2 days (range 1–4 days). The median number of needles inserted during ISBT was 15 (range 7–23).

**Table 2:** Treatment characteristics

Radiotherapy	
EBRT plus ISBT	41
ISBT alone	6
Anesthesia for ISBT	
Spinal and epidural anesthesia	32
Spinal anesthesia and IVPCA	15
Median duration of bed rest, day (range)	2 (1–4)
Median number of inserted needles, needle (range)	15 (7–23)
Mean volume of CTV (cc)	54.9 ± 32.4
Mean EQD2 for CTV and OARs (Gy)	
CTV D90 (α/β = 10)	71.0 ± 6.7
Bladder D2cc (α/β = 3)	61.7 ± 15.6
Sigmoid D2cc	44.2 ± 17.1
Rectum D2cc	58.5 ± 11.9

*EBRT* external beam radiotherapy, *ISBT* interstitial brachytherapy, *IVPCA* intravenous patients-control anesthesia, *CTV* clinical target volume, *EQD2* equivalent dose in 2 Gy fractions, *OAR* organ at risk

**Fig. 2:**
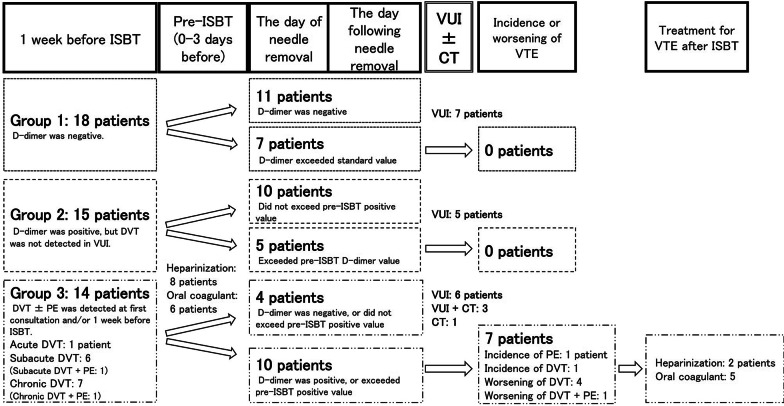
Incidence and worsening of venous thromboembolism after interstitial brachytherapy in each group. *ISBT* interstitial brachytherapy, *VUI* venous ultrasound imaging, *CT* computed tomography, *VTE* venous thromboembolism, *DVT* deep venous thrombosis, *PE* pulmonary embolism

VTE and DD status during ISBT, according to the patients’ assigned group, is shown in Fig. 2. There were no VTEs detected before ISBT in 33 patients. Of these, 18 patients were in group 1, and 15 patients were in group 2. In group 3, 12 of 14 patients with VTE before ISBT had DVT alone, and the remaining two patients had both DVT and PE. Of the 14 patients in group 3, VTE was detected at first consultation in eight patients and was detected 1 week before ISBT in six patients. In the VUI findings, 1 week before ISBT, one patient was diagnosed with acute DVT, six patients were diagnosed with subacute DVT, and seven patients had chronic DVT. Heparin was administered to eight patients, including seven patients with acute or subacute DVT and one patient with PE and chronic DVT. VUI ± CT was performed after needle removal in 22 patients. Of these, 18 patients received VUI alone, three received VUI and CT, and one received CT alone. VUI was omitted in this patient because DD levels were extremely high on the day of needle removal. We observed no incidence of VTE related to ISBT in 33 patients in groups 1 and 2. Among the 14 patients in group 3, we observed an incidence and/or worsening of VTE in seven patients. None of these seven patients had symptomatic VTE. In one patient, heparin administration was initiated 3 h after needle removal due to new presentation of VTE in multiple sites, including the lungs, and the DD level was extremely high (88.9 µg/mL). Another patient, in whom VTE extended to the inferior vena cava, was administered heparin on the day following needle removal. The remaining patients in group 3 received oral anticoagulants. During or after ISBT, none of the patients experienced bleeding requiring treatment.

**Fig. 3:**
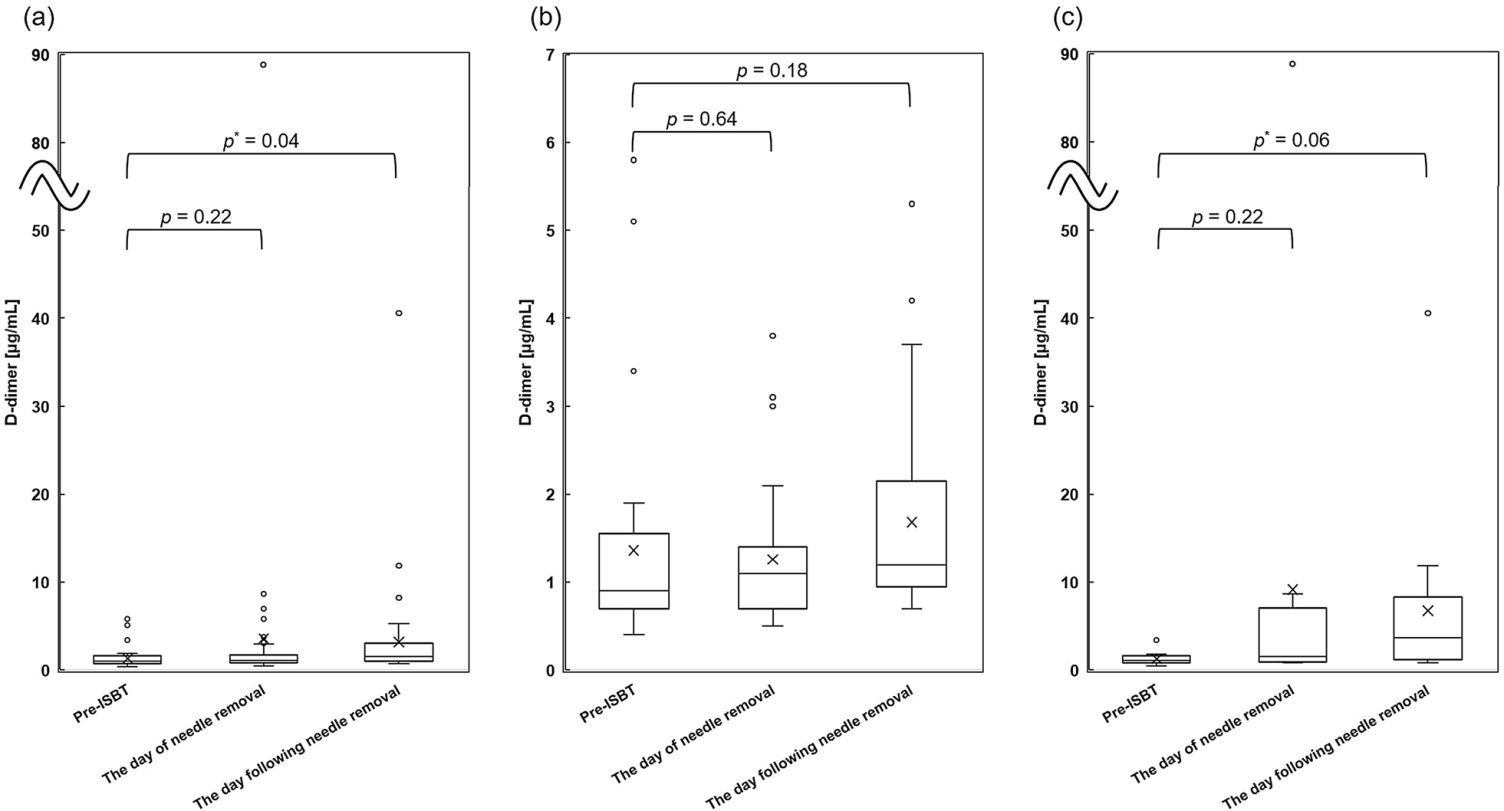
D-dimer values. D-dimer values pre-interstitial brachytherapy, on the day of and day following needle removal for all patients (**a**), values in patients without venous thromboembolism before interstitial brachytherapy (Groups 1 and 2) (**b**) and values in patients with venous thromboembolism before interstitial brachytherapy (Group 3) (**c**)

The DD values obtained pre-ISBT, on the day of, and on the day following needle removal are shown in Fig. 3. For all patients, the median pre-ISBT DD value was 1.0 µg/mL (range 0.4–5.8 µg/mL), the median value on the day of needle removal was 1.1 (0.5–88.9), and the median value on the day following needle removal was 1.5 (0.7–40.6). For all patients, DD values on the day following needle removal were significantly higher than those measured pre-ISBT (*p* = 0.04); however, there was no significant difference in DD values between the pre-ISBT levels and those on the day of needle removal (*p* = 0.22). The median difference in DD values between the pre-ISBT level and the day of needle removal was 0.0 µg/mL (− 4.4 to 85.5 µg/mL). For patients without VTE before ISBT, the median difference was 0.0 (− 4.4 to 3.1), for those with VTE, it was 0.2 (− 0.5–85.5), and among seven patients without and seven with incidence or worsening of VTE, the median difference was − 0.2 (− 0.5 to 0.4) and 6.1 (− 0.1 to 85.5), respectively. There was no significant difference between patients without VTE before ISBT vs. those with (*p* = 0.21) and between patients without incidence or worsening of VTE vs. those with (*p* = 0.22). The median difference between pre-ISBT DD values and the day following needle removal among all patients was 0.3 µg/mL (− 3.3 to 37.2 µg/mL), while among patients without VTE before ISBT, the median difference was 0.2 (− 3.3 to 4.6), and among patients with VTE, it was 2.0 (− 0.2 to 37.2). Among seven patients without and seven with incidence or worsening of VTE, the median difference was 0.1 (− 0.2 to 2.9) and 7.7 (1.3 to 37.2), respectively. There was a borderline significant difference between patients without VTE before ISBT vs. those with (*p* = 0.07) and between patients without incidence or worsening of VTE vs. those with (*p* = 0.07).

The univariate analysis revealed that the incidence or worsening of VTE was significantly related to a diagnosis of DVT before ISBT (*p* < 0.01), a Caprini score ≥ 7 (*p* < 0.01), a difference in DD values between pre-ISBT and the day of needle removal ≥ 1 (*p* < 0.01), and a difference between DD values pre-ISBT and on the day following needle removal ≥ 1 (*p* < 0.01) (Table 3). However, body mass index (BMI), longer duration of bed rest, and Rogers score were not significantly associated with the incidence or worsening of VTE (*p* = 0.27, 0.08, and 0.40, respectively).

**Table 3:** Univariate analysis for the incidence or worsening of venous thromboembolism

	Patients	Incidence or worsening of VTE	*p*-value
Age			
< 61 years	23	2	0.23
≥ 61 years	24	5	
Primary			
Cervix	37	7	0.16
Others	10	0	
Body mass index			
> 22 kg/m^2^	29	3	0.27
≤ 22 kg/m^2^	18	4	
Tumor size			
< 61 mm	23	3	0.53
≥ 61 mm	24	4	
PLN metastasis			
Negative	17	3	0.50
Positive	30	4	
PALA metastasis			
Negative	35	6	0.42
Positive	12	1	
Initial onset/recurrence			
Initial onset	33	5	0.65
Recurrence	14	2	
Duration of bed rest			
≤ 2 days	34	3	0.08
≥ 3 days	13	4	
Number of inserted needles			
≤ 15	29	4	0.55
≥ 16	18	3	
Volume of CTV			
≤ 54.9 cc	24	3	0.48
> 54.9 cc	23	4	
VTE before ISBT			
Negative	33	0	< 0.01
Positive	14	7	
Caprini score			
≤ 6	33	1	< 0.01
≥ 7	14	6	
Rogers score			
≤ 4	31	6	0.40
≥ 5	16	1	
Difference of D-dimer value between pre-ISBT and the day of needle removal			
< + 1 µg/mL	41	2	< 0.01
≥ + 1	6	5	
Difference of D-dimer value between pre-ISBT and the day following needle removal			
< + 1 µg/mL	34	0	< 0.01
≥ + 1	13	7	

*VTE* venous thromboembolism, *SCC* squamous cell carcinoma, *PLN* pelvic lymph node metastasis, *PALA* para-aortic lymph node, *CCRT* concurrent chemoradiotherapy, *RT* radiotherapy, *EBRT* external beam radiotherapy, *ISBT* interstitial brachytherapy, *CTV* clinical target volume, *DVT* deep vein thrombosis

## Discussion

It has been previously reported that without thromboprophylaxis, the incidence of symptomatic VTE following major surgery was 1.5–6.0% [15, 16]. In surgical patients with mechanical prophylaxis, administration of heparin, or both of these, the incidence of symptomatic VTE was 0.9–3.4%, 0.5–1.9%, or 0.2–0.9%, respectively [15–19]. These studies also reported that major bleeding was observed in 1.2–4.5% of patients [15, 17, 18]. The incidence of fatal PE in the patients with thromboprophylaxis was reduced to 0.1–0.5%, compared to 0.3–1.2% in the patients without thromboprophylaxis [4]. In the clinical practice guidelines reported by the American College of Chest Physicians and the American Society of Clinical Oncology, the type of thromboprophylaxis was determined according to the risk stratification of VTE and bleeding complications. For patients with a moderate risk of VTE, administration of heparin or mechanical prophylaxis was recommended, while for the patients at a high risk of VTE, the administration of heparin with the addition of IPCD was recommended [4, 20]. However, if the patients were at high risk for major bleeding complications, the administration of heparin was avoided. In this study, all patients were classified as moderate or high risk, as evaluated by the Caprini score. Heparin was only administered to patients with acute or subacute DVT or with PE before ISBT. This was because ISBT, in which 10 or more needles were placed for 2–3 days, was considered to be a procedure that had a potential for major bleeding. As a result, the incidence of VTE was observed in two (4.2%) out of 47 patients, and worsening of VTE was observed in five (10.6%) out of 47 patients. These results were similar to the incidence of symptomatic VTE following major surgery without thromboprophylaxis [15, 16]. However, the majority of previous reports evaluated the incidence of symptomatic VTE following surgery with or without thromboprophylaxis, not non-symptomatic VTE. In this study, symptomatic VTE was not observed, and no major bleeding complications occurred during or after ISBT under thromboprophylaxis.

The risk of VTE appeared to be highest for patients undergoing abdominal or pelvic surgery for malignancy [4, 21, 22]. In addition, advanced age (especially ≥ 60 years), prior VTE, bed rest (≥ 4 days), and longer length of hospital stay were also considered to be risk factors for VTE following surgery [4, 16, 23–25]. Tasaka et al. reported that the risk factors for VTE at pretreatment were advanced age (≥ 60 years) and bulky tumors (≥ 40 mm) for cervical cancer patients, and advanced age, stage III/IV disease, and bulky tumors (≥ 60 mm) for endometrial cancer patients [26]. Martin et al. reported that the incidence of VTE after surgery was significantly higher in patients with a BMI of ≥ 40 kg/m^2^ [27]. In the univariate analysis of this study, advanced age, BMI, bulky tumors, and long bed rest were not significantly related to the incidence or worsening of VTE (Table 3). However, the presence of VTE before ISBT, a Caprini score ≥ 7, a difference in DD values between pre-ISBT and the day of needle removal ≥ 1, and a difference between pre-ISBT and the day following needle removal ≥ 1 were risk factors for VTE. It was difficult to evaluate the association between the incidence or worsening of VTE during ISBT and family history of VTE because no patient had a family history of VTE. BMI was also difficult to evaluate because no patient had extremely high BMI in our study.

The risk stratification for VTE following surgery was mainly evaluated according to the Rogers [4, 8] and the Caprini scores [4, 9]. The Rogers score was established based on the analysis of VTE incidence in 183,069 patients undergoing vascular and general surgery [8] and consists of patient factors (female sex, higher American Society of Anesthesiologists class, ventilator dependence, preoperative dyspnea, and transfusion in the 72 h before operation), preoperative laboratory values (albumin, bilirubin, sodium, and hematocrit), and surgical characteristics (type of surgical procedure, work relative value units, and infected/contaminated wounds). In this study, all patients were categorized as very low risk because the Rogers score is composed of factors that are less affected by ISBT. In contrast, the Caprini score, which was mainly established by referring to the medical literature, contains patient factors (age, BMI, prior VTE, family history of VTE, coagulation factors, such as Factor V, Reiden and Lupus anticoagulant, malignancy, and bed rest) and surgical characteristics (type of surgical procedure). Shi et al. reported that the incidence of non-symptomatic VTE after surgery was 0%, 4.2%, and 50.0% for a Caprini score ≤ 5, 5–6, ≥ 7, respectively, in the gynecological cancer patients with thromboprophylaxis [28]. In this study, the incidence or worsening of VTE was significantly higher (43%) in the high-risk group with a Caprini score of ≥ 7 than in the high-risk group with a Caprini score of 5–6 (3%) or the moderate-risk group with a Caprini score of 3–4 (0%) (*p* < 0.01). In each risk group determined by Caprini score, the incidence of non-symptomatic VTE in our study was similar to that of gynecological cancer patients after surgery. Furthermore, if a Caprini score was ≥ 7, the incidence of non-symptomatic VTE was extremely high, and strict caution is required. The Caprini score was more suitable than the Rogers score to evaluate the risk for the incidence or worsening of VTE during ISBT because factors, such as age, bed rest, and prior VTE were included in the Caprini score.

To predict the incidence or worsening of VTE during ISBT, changes in DD values detected by close monitoring and a diagnosis of VTE before ISBT are necessary to screen gynecological cancer patients treated with ISBT. The Caprini score may be useful to predict the incidence or worsening of VTE, not only for the surgical patients but also for the patients treated with ISBT. Additionally, the close monitoring of DD values and the management of VTE that was modeled in this study could be suggested as a novel approach for thromboprophylaxis in gynecological cancer patients treated with ISBT.

The limitations of this study include its single-center retrospective design and small sample size. Thus, further studies with multicenter prospective designs and large sample sizes are required to establish the efficacy of screening and thromboprophylaxis for VTE during ISBT.

## Conclusions

DD levels should be closely measured before and after ISBT to detect the incidence or worsening of VTE for patients diagnosed with DVT before ISBT. The Caprini score may be useful to evaluate the risks for the incidence or worsening of VTE induced by prolonged bed rest during ISBT.

## Data Availability

The datasets supporting the conclusions of this article are available upon reasonable request.

## Abbreviations

VTE: Venous thromboembolism
DD: D-dimer
PE: Pulmonary embolism
ISBT: Interstitial brachytherapy
LC: Local control
HDR: High dose rate
VUI: Venous ultrasound imaging
DVT: Deep vein thrombosis
aPTT: Activated partial thromboplastin time
EBRT: External beam radiotherapy
MB: Midline block
HRCTV: High-risk clinical target volume
CCRT: Concurrent chemotherapy with radiotherapy
OAR: Organ at risk
EBRT: External beam radiotherapy
